# Fibroblast transformation in the tumor microenvironment of lung adenocarcinoma: heterogeneity, regulation, and therapeutic targeting

**DOI:** 10.3389/fimmu.2026.1786697

**Published:** 2026-05-05

**Authors:** Yujian Zhang, Yu Zhang, Jianhang You, Zhong Lu, Tao Zhao

**Affiliations:** 1 School of Clinical Medicine, Shandong Second Medical University, Weifang, Shandong, China; 2 Department of Oncology, Affiliated Hospital of Shandong Second Medical University, Weifang, Shandong, China; 3 Department of Central Laboratory, The People’s Hospital of Rizhao, Rizhao, Shandong, China; 4 Shandong Provincial Key Medical and Health Laboratory of Perioperative Precise Anesthesia and Organ Protection Mechanism Research, The People’s Hospital of Rizhao, Rizhao, Shandong, China; 5 Rizhao Key Laboratory of Basic Research on Anesthesia and Respiratory Intensive Care, The People’s Hospital of Rizhao, Rizhao, Shandong, China

**Keywords:** cancer-associated fibroblasts, heterogeneity, immunotherapy, lung adenocarcinoma, targeted therapy, tumor microenvironment

## Abstract

Cancer-associated fibroblasts (CAFs) are important stromal cells in the tumor microenvironment of lung adenocarcinoma (LUAD). This review discusses the different cellular sources of CAFs and the main signaling pathways involved in fibroblast-to-CAF transition, with attention to the biological settings that are especially relevant in LUAD. It further discusses CAF heterogeneity not only in terms of functional subtypes, but also from the perspective of dynamic cell states shaped by spatial niche, intercellular communication, and therapy-related stress. On this basis, we outline how distinct CAF states contribute to extracellular matrix remodeling, metabolic reprogramming, invasion and metastasis, angiogenesis, immunosuppression, and therapeutic resistance in LUAD. Particular attention is given to the treatment relevance of CAFs in driver-defined disease settings, where stromal programs may influence responses to targeted therapy and immunotherapy in a context-dependent manner. We also address the main obstacles to CAF-targeted strategies, particularly heterogeneity, plasticity, and functional compensation, and point to possible directions based on precise subtyping, functional modulation, and rational combination therapy. This review thus offers a LUAD-specific perspective for understanding CAF biology and for guiding more precise stromal intervention strategies.

## 1 Introduction

Fibroblasts, the primary cell type in connective tissue, are tasked with synthesizing collagen, elastin, and other extracellular matrix (ECM) components. This synthesis is crucial for maintaining the structural integrity of tissues and facilitating the repair of injuries (1, 2). Within the tumor microenvironment (TME), CAFs become activated in response to signals from tumor cells, together with the cytokines and metabolites released by these cells (3). Once activated, they differ clearly from normal fibroblasts in morphology, phenotype, and function. Their role shifts from maintaining tissue homeostasis to promoting tumor progression (4). CAFs are closely involved in tumor development and metastatic spread through their effects on extracellular matrix remodeling and the immune microenvironment (5). In lung adenocarcinoma (LUAD), these cells are increasingly viewed as major regulators of the TME. Defining their roles in LUAD more clearly may improve our understanding of disease progression and therapeutic resistance within this specific disease setting.

The leading cause of cancer-related death worldwide is LUAD, one of the most common subtypes of non-small cell lung cancer (6). The prognosis of patients with advanced LUAD remains poor despite continuous advancements in several therapeutic modalities, including surgery, chemotherapy, radiation, targeted therapy, and immunotherapy. One significant contributing factor is immunosuppression caused by TME (7). Among the most prevalent stromal cells in the TME, CAFs are engaged in the production of several growth factors and immune-regulatory factors in addition to tissue healing and ECM remodeling. By directly affecting the migration, invasion, and proliferation of cancer cells, these actions play a crucial role in regulating the development of LUAD (8). However, the molecular mechanisms through which CAFs shape LUAD progression, immune remodeling, and treatment resistance remain incompletely understood (9). Given the marked molecular heterogeneity and distinctive TME of LUAD, the roles of CAFs in this disease warrant discussion in a more specific context. A more focused examination of CAF biology in LUAD is therefore important for clarifying disease mechanisms and identifying potential therapeutic targets (10).

This review summarizes the origins, transformation mechanisms, heterogeneity, and functional diversity of CAFs in LUAD, with particular emphasis on their roles in stromal remodeling, immune regulation, and therapeutic resistance. It also discusses the major challenges facing CAF-targeted strategies and highlights potential future directions for more precise stromal intervention.

## 2 Transformation of fibroblasts into CAFs

The conversion of fibroblasts into CAFs is a key step in the remodeling of the LUAD TME (9). After this transition, CAFs undergo clear changes in morphology, phenotype, and function. These cells grow in size, adopt a spindle-shaped form, and contain ample cytoplasm. Molecularly, the transformed CAFs show enhanced expression of markers like vimentin and fibroblast activation protein (FAP). FAP participates in extracellular matrix remodeling and regulates certain bioactive peptides (11). By changing the mechanical properties and composition of the surrounding matrix, it facilitates tumor cell adhesion and migration (11). Through this shift from a quiescent state to an activated state, fibroblasts move from maintaining tissue stability to supporting tumor progression (12, 13).

CAFs frequently have diverse cell population origins, and their origin exhibits a high degree of heterogeneity (12, 14). In the LUAD microenvironment, CAFs typically transform from tissue-resident fibroblasts, bone-marrow-derived mesenchymal stem cells, adipocytes, epithelial cells, or endothelial cells (9, 13). This multi-source origin serves as the basis for their functional diversity (14).

Among the many regulatory factors involved, transforming growth factor-β (TGF-β) is regarded as a core signaling pathway driving CAF formation. TGF-β binds to its receptor, thereby activating the Smad signaling pathway. This results in the phosphorylation of the Smad2/3 protein (15). Phosphorylated Smad2/3 then binds Smad4 and forms a transcriptional complex that enters the nucleus. This increases α-smooth muscle actin (α-SMA) expression in fibroblasts. α-SMA gives CAFs strong contractile properties, which raise local matrix stiffness and tension, promote ECM synthesis, and drive tumor matrix remodeling (16).

Moreover, TGF-β can also interact with the Wnt/β-catenin pathway and further promote CAF activation. Vimentin is associated with maintenance of cell shape, intracellular signal transduction, and substance transport, thereby enhancing the migratory capacity of CAFs (17, 18). In conjunction with TGF-β, platelet-derived growth factor (PDGF) stimulates its receptor on fibroblasts. This, in turn, activates the PI3K/Akt and Ras/MAPK pathways, promoting fibroblast migration and proliferation (19).

They produce higher levels of fibronectin and collagen types I and III, and they also release matrix-degrading enzymes, including matrix metalloproteinases (MMP-2 and MMP-9), which accelerate ECM turnover and remodeling and thereby facilitate tumor cell invasion (20). CAFs also play an important part in establishing an immunosuppressive microenvironment. Activated CAFs secrete interleukin-6 (IL-6), which binds to the IL-6 receptor on the surface of T cells and activates the JAK-STAT3 signaling pathway (21). Persistently activated STAT3 suppresses the cytotoxic activity of effector T cells, promotes T-cell exhaustion, and obstructs T-cell infiltration (22). Furthermore, CAFs secrete interleukin-10 (IL-10), colony-stimulating factors, and chemokines. These secreted substances recruit immature myeloid cells from the bone marrow to the tumor site, inducing their differentiation into immunosuppressive myeloid-derived suppressor cells (MDSCs) and facilitating the accumulation of M2 macrophages (23, 24).

M2 macrophages lack cytotoxic function; instead, they secrete increased amounts of IL-10, which intensifies immunosuppression and promotes angiogenesis. M2 macrophages secrete substantial amounts of immunosuppressive substances, as do recruited and polarized MDSCs. Collectively, these factors establish an immunosuppressive milieu that weakens the host’s anti-tumor response, enabling tumor cells to elude immune surveillance (25). In the LUAD microenvironment, CAFs are pivotal in tumor growth and immune evasion by remodeling the tumor stroma and regulating immune responses.

**Figure 1:**
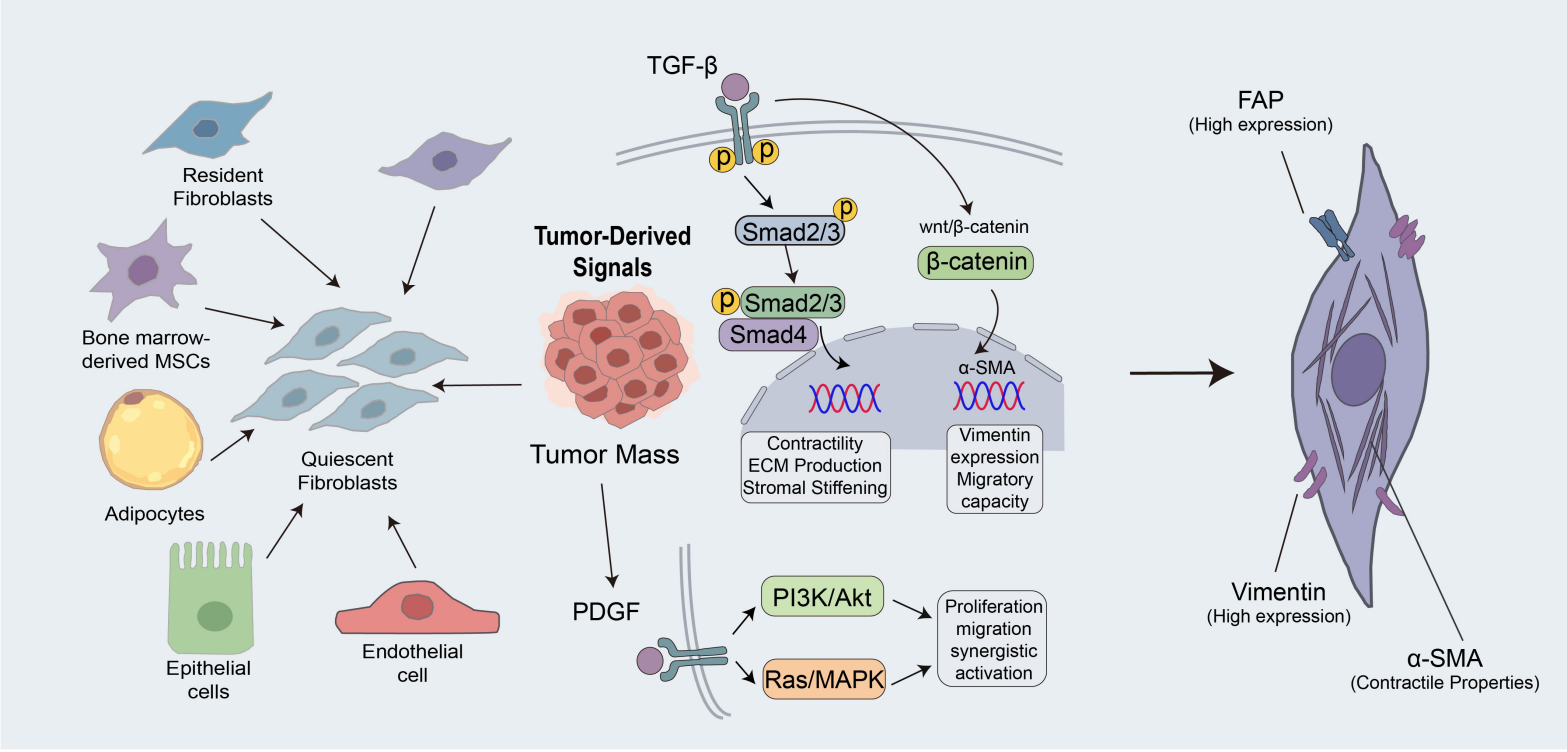
Transition of fibroblasts into cancer-associated fibroblasts (CAFs) in LUAD. This figure illustrates the cellular origins, key signaling pathways, and consequences of fibroblast-to-CAF transition in LUAD. Tumor-derived signals such as TGF-β and PDGF drive fibroblast activation, extracellular matrix remodeling, and immune suppression, establishing CAF heterogeneity at early stages of tumor microenvironment remodeling.

It is noteworthy that different induction signals and cell origins collectively determine the direction and outcome of fibroblast transformation into CAFs. This process does not lead to a single endpoint but initiates the formation of a CAF population with multiple subtypes and functions. In other words, CAF heterogeneity is established early in the transformation, and this heterogeneity is a major source of the complexity of the LUAD TME (26) (**Figure 1**).

## 3 CAF heterogeneity

The CAF population is highly heterogeneous, consisting of multiple subtypes that exhibit distinct molecular signatures, spatial distributions, and functional roles (27). This heterogeneity stems not only from their diverse cellular origins but also from the signaling stimuli within the TME (28).

Recent single-cell and spatial transcriptomic studies have changed this view, suggesting that CAF heterogeneity in LUAD should not be understood only as a set of discrete subtypes defined by markers, but more as a group of dynamic, context-dependent cell states distributed across different spatial niches, with distinct CAF phenotypes showing non-random localization and links to specific TME features and clinical outcomes (31). From this perspective, CAF heterogeneity is better understood by considering cell state, spatial niche, and intercellular communication together, rather than relying only on fixed subtype boundaries.

In LUAD, several functionally specialized CAF subtypes have been identified, and each has a different role in immune regulation, matrix remodeling, and signal transduction (29).

One of the earliest recognized subtypes is myofibroblastic cancer-associated fibroblasts (myCAFs), whose biological characteristics are similar to those of activated fibroblasts during wound healing. myCAFs are characterized by the upregulated expression of α-smooth muscle actin (α-SMA), fibroblast activation protein (FAP), and extracellular matrix-related genes. They participate in stromal remodeling, extracellular matrix deposition, and tissue stiffening, thereby promoting tumor progression (30). In addition, single-cell sequencing and spatial transcriptomic analyses have identified additional cancer-associated fibroblast subpopulations related to lung cancer, such as POSTN^+^ cancer-associated fibroblasts and transitional CXCL14^+^ myofibroblastic cancer-associated fibroblasts. These subpopulations exert multiple functions in the tumor microenvironment and are involved in processes including immunosuppression, tumor progression, metastasis, and resistance to epidermal growth factor receptor tyrosine kinase inhibitors (EGFR-TKIs) (31, 32).

Another important subtype is inflammatory CAFs (iCAFs), which are enriched for cytokines and chemokines such as IL-6, CXCL1, and CXCL12 and are thought to participate in immune regulatory circuits in a context-dependent manner, suggesting that inflammatory CAF programs should be interpreted not as a fixed functional category, but as a plastic state shaped by the surrounding immune and stromal microenvironment (33). After radiotherapy, DNA damage and cytokine release activate the transcription factor IRF1 (Interferon Regulatory Factor 1), prompting some iCAFs to transform into ilCAFs, exhibiting dual immune characteristics (34). ilCAFs, in turn, highly express IFN-β, CXCL9, and CXCL10. These mediators recruit cytotoxic T cells and reshape the post-radiotherapy immune microenvironment, thereby influencing treatment response (35).

Antigen-presenting CAFs (apCAFs) have emerged as an important subtype in recent CAF research. apCAFs express major histocompatibility complex II (MHC II) molecules but lack co-stimulatory molecules, and are mainly distributed in the tumor stroma or peripheral regions (36). Their MHC II expression can be upregulated by IFN-γ, TNF-α, oxidative stress, and IL-1 within the TME. They can present antigens, but due to the lack of a second signal, they often induce CD4^+^ T cell inactivation or differentiation into regulatory T cells (Tregs), thereby forming an immunosuppressive microenvironment (33). However, under signals like C1q-C1qbp, apCAFs can also support anti-tumor CD4^+^ T cells, demonstrating functional duality (37). Their spatial distribution, inducing factors, and interaction with radiotherapy/chemotherapy—especially the significant enhancement of IFN-γ after radiotherapy—may make them important regulators of tumor prognosis and immune therapy response (38).

**Figure 2:**
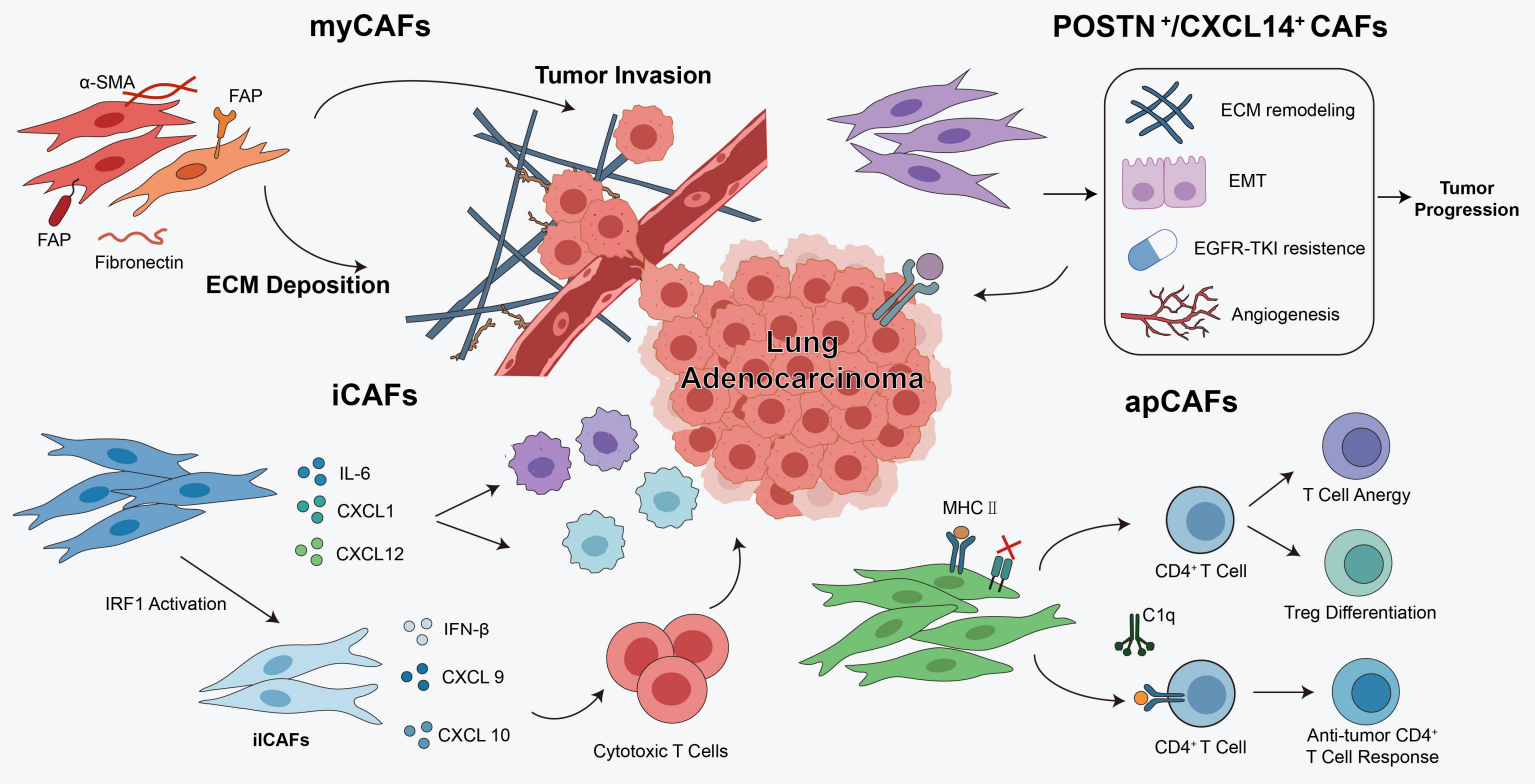
CAF heterogeneity and functional plasticity in LUAD. This figure illustrates the heterogeneity and functional plasticity of CAFs in LUAD. Distinct CAF subtypes exhibit specialized roles in extracellular matrix remodeling, angiogenesis, immune regulation, and therapeutic resistance, collectively shaping the tumor microenvironment and influencing disease progression and treatment response.

Overall, a more refined classification of CAF states in LUAD is important for understanding the complexity of the TME, identifying specific biomarkers, and guiding the development of precise and effective CAF-targeted therapies (**Table 1**, **Figure 2**).

**Table 1:** Key characteristics of major CAF subtypes in LUAD.

CAF subtype	Representative markers	Inducing factors/Context	Spatial distribution	Core functions in LUAD
myCAFs	α-SMA, FAP, extracellular matrix-related genes; LUAD-relevant states include POSTN^+^ CAFs and transitional CXCL14^+^ myofibroblastic CAFs (31, 32)	TGF-β, matrix tension, and wound-healing-like activation programs	Stromal and fibrotic regions; often enriched near the invasive front	Mediate stromal remodeling, extracellular matrix deposition, and tissue stiffening, thereby facilitating tumor progression; LUAD-relevant POSTN^+^ and CXCL14^+^ states are additionally associated with immune suppression, metastasis, and resistance to EGFR-TKIs
iCAFs	IL-6, CXCL1, CXCL12; under radiotherapy/IFN-related stress may acquire IFN-β, CXCL9, and CXCL10 expression	Inflammatory signals such as IL-1; NF-κB/JAK-STAT-related programs; context-dependent immune cues; radiotherapy-induced stress and IRF1-associated interferon signaling can drive transition toward an ilCAF-like state	Immune cell-infiltrated stromal regions (33)	Participate in immune regulatory circuits and inflammatory signaling; can promote immunosuppressive microenvironment formation in a context-dependent manner; after radiotherapy, may recruit cytotoxic T cells while also contributing to tumor adaptation to radiotherapy
apCAFs	MHC II, C1q-related features	IFN-γ, TNF-α, oxidative stress, and IL-1	Tumor stroma or peripheral stromal regions (36)	Exhibit functional duality: may induce CD4^+^ T-cell inactivation or Treg differentiation because of deficient co-stimulation, but can also support anti-tumor CD4^+^ T-cell responses through C1q-C1qbp signaling (33)

## 4 Role of CAFs in lung adenocarcinoma progression

The marked heterogeneity of CAFs means that they can exert complex and sometimes even opposing effects in LUAD (39). As major regulators in the TME, CAFs shape LUAD progression through multiple routes, including promoting tumor cell proliferation, enhancing invasion and metastasis, inducing angiogenesis, facilitating immune evasion, and aggravating therapeutic resistance (40). This section discusses the main pro-tumor and anti-tumor mechanisms mediated by different CAF subtypes and provides relevant evidence and new ideas for the precise treatment of LUAD.

### 4.1 Promoting tumor cell proliferation and metabolic reprogramming

CAFs mainly rely on secreting various growth factors and exosomes to provide core support for tumor cell proliferation (17). CAFs are capable of secreting epidermal growth factor (EGF) and fibroblast growth factor-2. These growth factors activate the MAPK/ERK and PI3K/Akt signaling pathways. As a result, the expression of cell-cycle proteins Cyclin D1 and Cyclin E is upregulated, cell-cycle progression is promoted, and tumor cell proliferation is accelerated (41). Additionally, CAFs inhibit the apoptosis pathways mediated by Bax and Caspase-3. By simultaneously promoting proliferation and inhibiting apoptosis, CAFs act synergistically to foster tumor growth (42, 43). Moreover, CAFs secrete insulin-like growth factor-1, which enhances the cancer cells’ capacity to uptake glucose and increases aerobic glycolysis activity. This provides energy support and satisfies the high-energy requirements of the tumor (44).

In LUAD, extracellular vesicles (exosomes) play a key role in regulating LUAD cell metabolism (45). Exosomes derived from CAFs carry miR-1290. This microRNA targets and inhibits the expression of the CUL3 gene. Consequently, the expression of glutamine transporters in LUAD cells is upregulated, thereby augmenting the tumor cells’ ability to uptake glutamine (46, 47). The large amount of glutamine taken up is used in the tumor cells’ tricarboxylic acid cycle to generate α-ketoglutarate and malate, ultimately promoting the production of ATP and NADPH (48). Glutamine acts as a nitrogen source, participating in the synthesis of purines, pyrimidines, and amino acids, providing sufficient energy and biosynthetic precursors for the rapid proliferation of cancer cells (47). The presence of exosomes further promotes tumor cell invasion and metastasis.

### 4.2 Promoting tumor invasion, metastasis, and angiogenesis

During the process of tumor invasion and metastasis, CAFs disrupt tissue barriers, reshape the microenvironment, and direct cancer cell migration via multiple mechanisms. CAFs release TGF-β1, which activates the Smad signaling pathway. This activation induces epithelial–mesenchymal transition (EMT), resulting in the down-regulation of E-cadherin and the up-regulation of N-cadherin and vimentin. Consequently, the migration and invasion capabilities of cancer cells are enhanced (49). Simultaneously, CAFs secrete MMP-2 and MMP-9. These enzymes degrade the original matrix, creating low-resistance migration channels for tumor cell infiltration (50, 51).

The high expression of CXCL14 in lung fibroblasts can remarkably enhance the tumor migration ability. Fibroblasts overexpressing CXCL14 upregulate genes associated with collagen degradation and matrix remodeling. This upregulation alters the structure of the extracellular matrix, leading to the degradation of collagen and extracellular matrix tissue. As a result, tumor cell proliferation and migration are promoted (32). Moreover, CAFs upregulate key oncogenes c-Myc, PIM1, and Cyclin D1 via the Wnt/β-catenin signaling pathway (52). Ligands such as CTHRC1 and Wnt2 cause β-catenin to accumulate in the cytoplasm. Subsequently, β-catenin translocates into the nucleus and initiates the transcription of downstream genes, which are direct β-catenin target genes. c-Myc promotes cell-cycle progression and suppresses p21, and Cyclin D1 directly mediates the G1 to S-phase transition of the cell. Together, these two processes accelerate tumor cell proliferation (53).

CAFs can also contribute to the formation of pre-metastatic niches at distant sites. There, they deposit ECM components and secrete factors to remodel the local microenvironment and induce angiogenesis, thereby facilitating metastatic colonization (54).

CAFs collaborate to promote tumor angiogenesis through multiple mechanisms, serving as a crucial nexus connecting tumor progression and microenvironment remodeling (55). CAFs secrete vascular endothelial growth factor (VEGF) and platelet-derived growth factor-BB, which stimulate the proliferation and migration of vascular endothelial cells, driving new blood vessel formation to fulfill the tumor’s requirements for oxygen and nutrients for growth (56, 57).

CAFs secrete the chemokine CXCL12, which binds to its receptor CXCR4 on the surface of cancer cells. This binding forms a chemoattractant gradient that guides cancer cells in their directional migration (58). Additionally, CXCL12 recruits endothelial progenitor cells to the local tumor area, contributing to the construction of new blood vessels. Simultaneously, it activates the VEGFR2 receptor present on vascular endothelial cells. This activation initiates the ERK and Akt signaling pathways, thereby facilitating endothelial cell proliferation and migration (59).

Furthermore, CAFs are capable of synthesizing laminin and fibronectin, offering structural support for tumor neovascularization. Besides, CAFs can differentiate into perivascular cells which envelop endothelial cells. This differentiation bolsters the continuity of the tumor blood supply and enhances vascular stability. Consequently, it promotes rapid tumor growth and metastasis (60).

### 4.3 Shaping an immunosuppressive microenvironment

CAFs create an immunosuppressive TME via diverse mechanisms, thereby emerging as a significant hurdle to immunotherapy (61). Through the secretion of numerous extracellular matrix components, CAFs build a dense physical barrier. This barrier effectively blocks the infiltration of cytotoxic T cells and natural killer cells into the tumor parenchyma, ultimately resulting in immune exclusion (62).

At the level of cellular regulation, CAF-derived chemokines such as CCL2 and CXCL12 recruit immunosuppressive cells, including Tregs and MDSCs. Cytokines such as IL-6 and TGF-β also support the survival, proliferation, and functional polarization of these cells within the microenvironment, helping to establish an immunosuppressive cellular network (63, 64).

At the molecular level, CAFs may express immune checkpoint molecules such as PD-L1 and PD-L2, which can contribute to T-cell dysfunction and exhaustion. CAF-associated indoleamine 2, 3-dioxygenase activity may likewise promote tryptophan depletion in the microenvironment and further weaken T-cell function (61, 65, 66).

In addition, CAFs also impair the maturation and antigen-presenting function of dendritic cells by secreting IL-10 and TGF-β (67, 68). TGF-β reduces the expression of MHC II molecules and co-stimulatory molecules, and it also upregulates the ID1 gene, which inhibits the differentiation of dendritic cells (DCs) into mature antigen-presenting cells and weakens their antigen-presenting capacity (69–71). Antigen presentation by dendritic cells depends on activation of the nuclear NF-κB signaling pathway, whereas TGF-β released by cancer-associated fibroblasts can inhibit this pathway and disrupt dendritic-cell maturation (72–74). TGF-β also limits the migration of dendritic cells to the lymphatic system, which further lowers the efficiency of antigen transport (70, 75). As a result, CAF-mediated suppression of dendritic-cell function weakens antigen presentation at multiple levels, interferes with the activation of CD4^+^ and CD8^+^ T cells, and ultimately impairs the initiation of adaptive immune responses (72, 76, 77).

By forming physical barriers, recruiting immunosuppressive cells, directly suppressing immune function, and disrupting antigen presentation, CAFs create a complex immunosuppressive microenvironment that reduces the effectiveness of immunotherapy.

### 4.4 Anti-cancer potential of CAFs

Despite the fact that the majority of CAFs display pro-cancer traits within the TME, their functionality is not uniform. Recent research has uncovered that certain subpopulations of CAFs have the potential to exert anti-cancer effects (78). Under particular conditions, these CAF subpopulations can bring about anti-cancer effects via diverse mechanisms, such as immune activation, exosome-mediated signaling, modulation of drug sensitivity, and matrix remodeling. This provides new ideas for clinical intervention strategies targeting CAFs (79).

apCAFs present tumor antigens to CD4^+^ T cells through the MHC II and secrete C1q. This secretion promotes the survival and activation of T cells, consequently inhibiting tumor growth. In LUAD, MHC II+ fibroblasts have been shown to sustain local CD4^+^ T-cell immunity through *in situ* antigen presentation and C1q-C1qbp signaling, indicating that apCAFs may, under specific conditions, exert immune-supportive rather than purely immunosuppressive functions (37). In the event of DNA damage or cellular stress, certain CAFs are capable of activating the intracellular STING-IRF3 signaling pathway. This activation induces the expression of Type I Interferons. These interferons then attract and activate CD8^+^ T cells, thereby augmenting the anti-tumor immune response and significantly impeding tumor progression (80). This mechanism suggests that drug-mediated regulation of the STING pathway can guide CAFs to transform from a pro-tumor to an anti-tumor phenotype, offering new ideas for therapeutic strategies (81). These observations suggest that the functional trajectory of apCAFs may be determined by the balance between co-stimulatory deficiency, inflammatory cues such as IFN-γ, C1q-C1qbp signaling, and therapy-induced stress within the local microenvironment.

**Figure 3:**
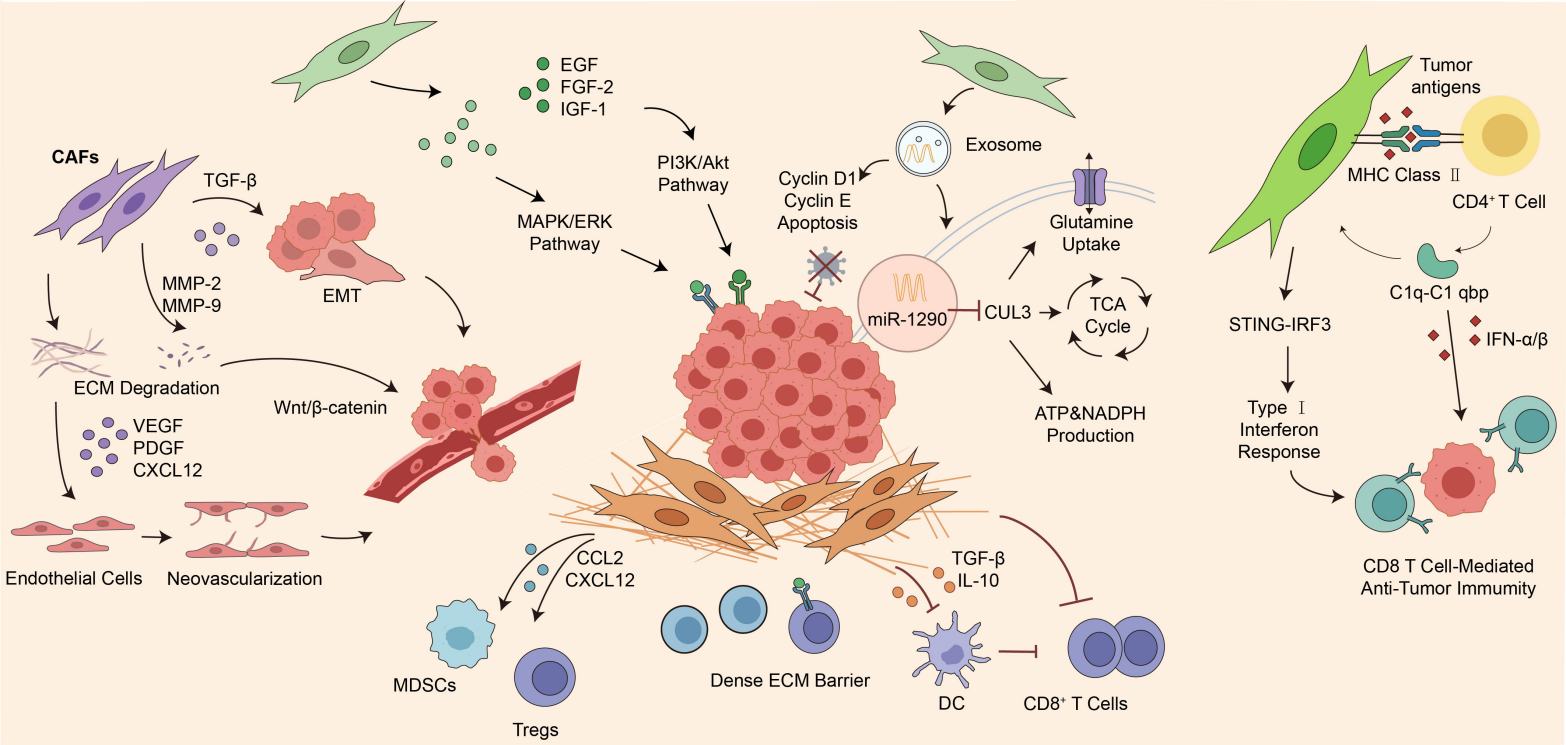
Multifaceted roles of cancer-associated fibroblasts (CAFs) in LUAD progression. This figure illustrates the multifaceted and context-dependent roles of CAFs in LUAD progression. CAFs regulate tumor growth, metabolism, invasion, angiogenesis, immune suppression, and therapeutic resistance, while specific CAF subsets exhibit anti-tumor potential by promoting immune activation. These findings highlight CAF heterogeneity as a critical determinant of tumor progression and therapeutic response.

Accordingly, clarifying how CAF heterogeneity shapes the immune landscape of LUAD, including through computational approaches such as CIBERSORT, could support more precise modulation of the tumor immune microenvironment and inform the development of more effective therapeutic strategies (82, 83). This entails both the targeted inhibition of pro-immunosuppressive CAF subtypes, thereby weakening their role in impeding immune cell infiltration and secreting immunosuppressive factors, and the exploitation of pro-immune-activating CAF subtypes to boost their capacity to recruit cytotoxic immune cells. The goal of this approach is to relieve tumor-associated immunosuppression, improve the efficacy of immunotherapy, and limit damage to normal tissues. Future treatment strategies targeting CAFs need to be tailored to specific contexts. They should move toward accurately distinguishing and targeting pro-tumor CAF subtypes while preserving or activating subtypes with anti-tumor potential, so that the TME can be regulated in a more precise way (**Figure 3**).

### 4.5 Mediating targeted therapy resistance

CAFs are important contributors to resistance to targeted therapies and represent a major clinical challenge in LUAD. Importantly, CAF-mediated resistance is not a generic stromal phenomenon, but rather a dynamic adaptation program that is highly dependent on specific oncogenic drivers (84). Emerging evidence indicates that CAF functions in LUAD are intricately linked to these driver-defined therapeutic contexts, shaping distinct resistance mechanisms.

In EGFR-mutant LUAD, CAF-mediated resistance is supported through metabolic, signaling, and physical mechanisms. Recent studies have shown that CAFs can maintain resistance to EGFR-TKIs through a CTHRC1-associated glycolysis–lactate positive feedback loop linked to histone lactylation (52). CAF-derived HGF can also activate c-MET signaling in tumor cells, allowing them to bypass EGFR inhibition and restore downstream PI3K/AKT and MAPK pathway activity (85). At the same time, CAF-driven ECM remodeling forms a collagen-rich and mechanically dense stroma, which raises interstitial pressure, reduces drug penetration, and helps residual tumor cells survive under therapeutic stress (86).

In ALK-rearranged LUAD, CAFs can promote resistance to ALK inhibition through both metabolic reprogramming and stromal signaling. One recent study showed that CAFs enhance *de novo* lipid biosynthesis in tumor cells under ALK-TKI treatment, whereas another found that CAFs confer resistance through concurrent integrin and MET signaling (84, 87). These findings indicate that resistance in ALK-driven LUAD may result from the joint action of stromal signaling and metabolic adaptation, rather than from a single bypass pathway.

In KRAS-mutant LUAD, stromal and inflammatory remodeling has also become an important feature. In this context, CAF-related inflammatory activation may help create a treatment-refractory microenvironment that supports tumor persistence and weakens effective anti-tumor immunity (88, 89). Integrated transcriptomic analyses further suggest that CAF-rich stromal states in LUAD are associated with immune exclusion, reduced cytotoxic lymphocyte infiltration, and poorer predicted benefit from immunotherapy, underscoring the contribution of both stromal composition and local fibroblast–immune cell interactions to CAF-mediated immunosuppression (83). Together, these changes may reinforce therapeutic tolerance and contribute to poor treatment responses.

Collectively, these driver-specific interactions underscore that CAF-mediated therapeutic resistance in LUAD is shaped by distinct molecular contexts. These context-dependent resistance programs provide an important basis for understanding the subsequent challenges of CAF-targeted therapy.

## 5 Challenges in CAF-targeted therapy

The central role of CAFs in LUAD is increasingly recognized, yet successfully translating them into effective therapeutic targets faces fundamental challenges (8, 90). These challenges mainly stem from the inherent high heterogeneity of CAFs, their dynamic phenotypic plasticity, and the resulting functional compensation effect (90). These three factors collectively constitute major barriers to CAF-targeted therapy, making “one-size-fits-all” treatment strategies ineffective (91). This section will elaborate on the challenges faced by CAF-targeted therapy.

The CAF population currently lacks universally applicable markers. Commonly used markers such as α-SMA and FAP do not fully capture the phenotypic and functional diversity of CAF states. Even within the same tumor, CAF subpopulations in different regions exhibit distinct phenotypes and functional characteristics. myCAFs are often enriched at the invasive front, whereas iCAFs are more frequently distributed in immune cell-infiltrated stromal regions. This spatial heterogeneity increases the difficulty of precise targeting and suggests that effective therapeutic strategies may need to account for specific microenvironmental niches (92–94).

Moreover, CAFs are not terminally differentiated cells; they can interconvert in response to signals in the microenvironment, exhibiting strong plasticity. Under therapeutic pressures like radiotherapy and chemotherapy, the composition of CAF subpopulations undergoes significant remodeling. After radiotherapy, the proportion of myCAFs decreases, while the proportion of iCAFs increases. iCAFs can progressively polarize into ilCAFs along an IFN-related program, accompanied by continuous upregulation of IRF1, forming a dynamic trajectory from myCAFs to iCAFs, and subsequently to ilCAFs (34). Inflammatory signals such as IL-1 activate the NF-κB and JAK/STAT pathways, inducing and maintaining the iCAF phenotype (95). Conversely, high concentrations of TGF-β inhibit JAK/STAT signaling and strongly activate the TGF-β/Smad and Rho/ROCK pathways, driving fibroblasts to differentiate into α-SMA-expressing myCAFs (96–98). This phenotype conversion induced by therapeutic pressure keeps the functional state of CAF subpopulations in a dynamic flux (34). Ultimately, the coexistence of inter-tumor and intra-tumor differences results in a highly uncertain therapeutic landscape, leading to limited or failed treatment efficacy (99, 100).

The functional complementarity and compensatory activation of different CAF subpopulations create an additional barrier to effective CAF-targeted intervention. When the myCAF pro-tumor subpopulation, which mediates ECM remodeling, is inhibited, other iCAF subpopulations that dominate immunosuppression may be activated or enhanced in a compensatory manner. They maintain or even strengthen tumor progression by driving M2 macrophage polarization and promoting Treg expansion (101). This functional difference and dynamic interconversion characteristics among different subpopulations within the same tumor form a multi-level therapeutic resistance network, which means that single-target strategies may not only be ineffective but could even trigger adverse effects through such compensatory mechanisms, ultimately leading to treatment failure.

**Figure 4:**
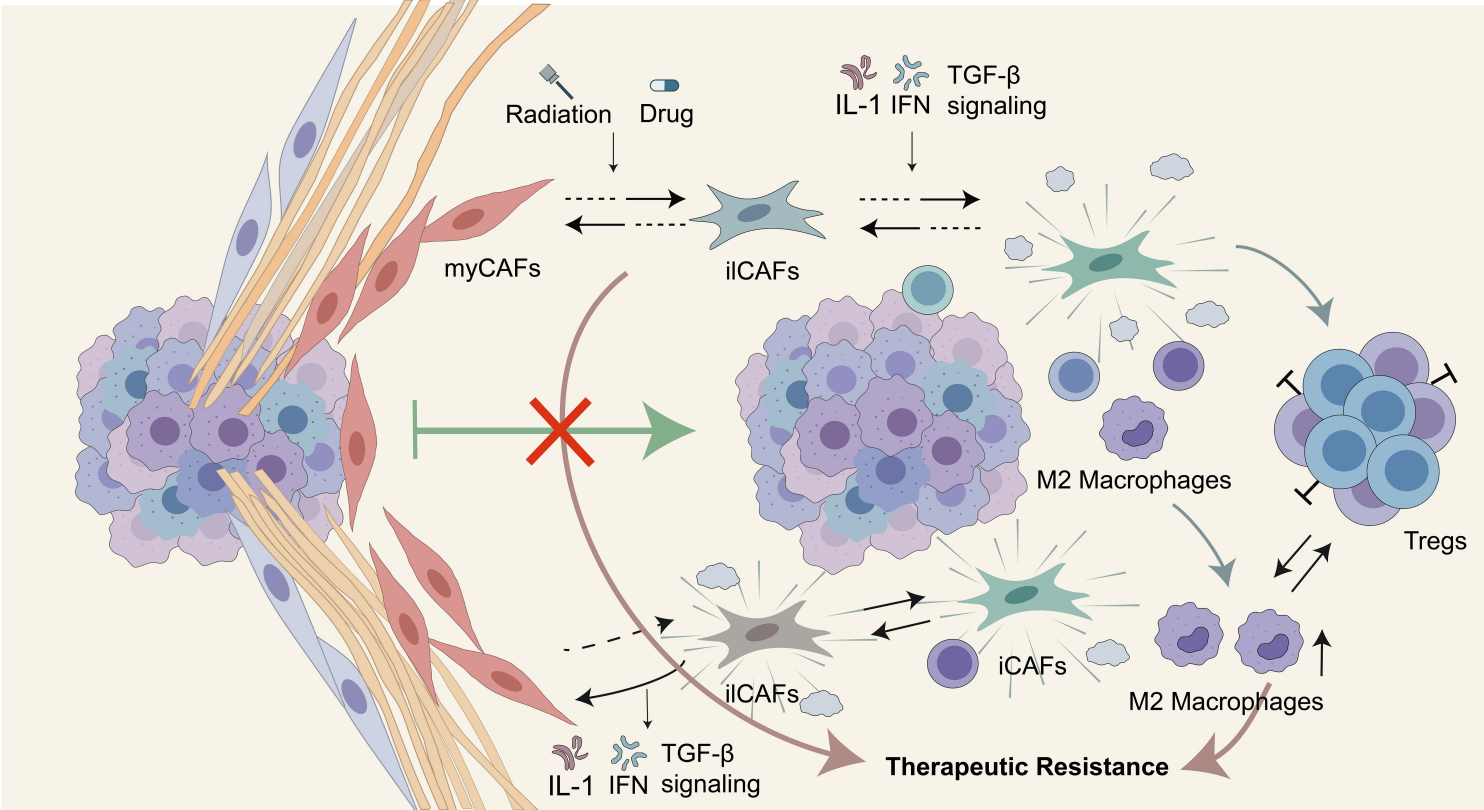
Major challenges in targeting CAFs. This figure summarizes the major challenges in targeting CAFs in LUAD. CAF heterogeneity, dynamic phenotypic plasticity, and functional compensation collectively limit the efficacy of conventional CAF-targeted therapies. Emerging precision strategies focusing on functional state modulation rather than nonspecific CAF depletion may provide more effective therapeutic opportunities.

Therefore, the key to overcoming the current bottleneck in targeted therapy lies in using cutting-edge technologies such as single-cell sequencing to deeply analyze the dynamic regulatory network of CAFs in both spatial and temporal dimensions. Based on this, precision strategies targeting specific functional states, rather than simply eliminating specific cell subpopulations, must be developed. Deep analysis of CAF heterogeneity and the elucidation of their dynamic regulation mechanisms are key prerequisites for overcoming the current bottleneck in targeted therapy (**Figure 4**).

## 6 Countermeasures

Faced with the therapeutic challenges posed by CAF heterogeneity, plasticity, and functional compensation, traditional broad-spectrum targeted strategies are proving insufficient. The future lies in shifting toward precise regulation: accurately identifying and intervening with specific pro-tumor subpopulations while preserving or activating anti-tumor subpopulations (102). This is not only to improve efficacy but also to avoid the potential counter-effects that may result from blindly clearing all CAFs (103).

### 6.1 Phenotype-specific targeting

An important approach is phenotype-specific targeting, which uses differences in marker expression among CAF subpopulations to selectively target pro-tumor fibroblasts within the TME. FAP is one of the most widely studied CAF markers and is highly expressed in LUAD-derived CAFs, whereas its expression in normal tissues is relatively limited, which makes it an attractive therapeutic target (104, 105). In preclinical human lung cancer xenograft and syngeneic mouse models, FAP-targeted stromal approaches have shown anti-tumor activity and can reduce tumor vessel density, inhibit abnormal fibroblast proliferation, and suppress tumor progression (106–108). These approaches selectively deplete FAP^+^ CAFs in the TME, which reduces extracellular matrix synthesis. The resulting relaxation of the ECM structure may improve vascular perfusion in tumor tissue and facilitate the infiltration of immune cells such as effector T cells and NK cells, thereby contributing to anti-tumor effects (101, 108).

Another distinct CAF subpopulation is CXCL14-positive myofibroblasts, which emerge at an early stage of myCAF differentiation and are referred to as transitional myCAFs because of their high CXCL14 expression. This subpopulation promotes EMT and angiogenesis, increases tumor invasiveness, and induces resistance to EGFR-TKIs, which can markedly affect therapeutic outcomes. CXCL14, as a key marker of this myCAF subpopulation, appears to be directly involved in its pro-metastatic and drug-resistant activities. In patients with lung cancer, including LUAD, higher plasma CXCL14 levels have been reported to correlate with more advanced clinical stage. Preclinical pharmacologic screening has identified filgotinib as a candidate agent that may counteract EGFR-TKI resistance mediated by transitional CXCL14^+^ myCAFs, suggesting that the CXCL14-associated program could represent a potential therapeutic vulnerability (32, 109).

Bispecific CAR-based strategies targeting both CAF markers and tumor antigens have also been explored. Available studies indicate that these CAF-targeted cellular therapies may increase IFN-γ secretion and reduce Treg abundance, although the evidence remains mainly at the preclinical stage (110, 111). FAP-targeted radioligand therapy is also under investigation as an approach that takes advantage of FAP specificity to deliver radioactive isotopes to the tumor stroma, with the aim of eliminating CAFs while limiting damage to normal tissues (112).

### 6.2 Functional heterogeneity targeting

Lactate generated by glycolytic CAFs serves as an important energy source for tumor cells (113). Metabolic reprogramming, such as aerobic glycolysis in CAFs, provides critical energetic support to tumor cells and contributes to a treatment-refractory microenvironment (114). Rather than eliminating CAFs, this strategy focuses on normalizing or restraining their core pro-tumor functions.

Monocarboxylate Transporter (MCT) inhibitors can block the transport of lactate from CAFs to tumor cells, leading to decreased ATP supply and an imbalance in the intracellular NAD+/NADH ratio, achieving a precise cutoff of the energy supply chain (103). Energy deprivation reduces the efficiency of the mitochondrial electron transport chain, generating excessive reactive oxygen species, which further exacerbates DNA double-strand breaks (115). Reactive oxygen species and energy deficiency collectively inhibit the expression and activity of key DNA repair proteins, making it difficult for radiation-induced DNA double-strand breaks to be quickly repaired, thereby increasing the cell death rate (116, 117).

Metabolic inhibition strategies require precise control of the inhibition intensity. Excessive blockage might lead to the transformation of CAFs towards a more invasive myofibroblast phenotype, which could instead enhance the tumor’s metastatic potential. Therefore, a dose gradient strategy should be designed considering CAF metabolic heterogeneity (26).

### 6.3 Immune microenvironment remodeling

Given the potential tumor-suppressing function of CAFs and the risk that complete removal of CAFs may exacerbate immunosuppression, reprogramming activated pro-cancer CAFs into a quiescent state has become a highly attractive new direction (108). The goal is to reshape activated CAFs, especially iCAFs, into a phenotype closer to the quiescent state, thereby inhibiting their pro-inflammatory and immunosuppressive functions and improving the delivery of chemotherapeutic drugs (81). In addition, strategies such as supplementing co-stimulatory signals, regulating the C1q-C1qbp axis, and modulating MHC II expression may help shift antigen-presenting CAFs (apCAFs) from an immunosuppressive state toward immune-supportive functions, thereby providing a potential basis for precision immunomodulatory therapy. The angiotensin receptor blocker losartan has been reported to modulate TGF-β-related stromal remodeling. It may reduce matrix fibrosis and improve vascular normalization, which can facilitate the penetration of chemotherapeutic drugs and immune cells (100, 107).

In addition to promoting CAF normalization, induction of a senescence-like stromal state has also been explored as a possible strategy. Prolonged CDK4/6 inhibition with abemaciclib can induce a p53-dependent senescent state in primary fibroblasts and *in vivo* models, suggesting that similar stromal changes may also occur in the TME (118). Senescence-associated changes in CAFs may further reshape the immune and stromal composition, although direct evidence that abemaciclib induces a comparable senescent state in LUAD-associated CAFs, followed by effective immune clearance, remains limited (119).

Multidimensional strategies such as TGF-β blockade and functional reprogramming of CAFs may therefore provide a practical way to reduce pro-tumor effects, improve drug delivery efficiency, and activate the tumor immune microenvironment in LUAD (**Table 2**).

**Table 2:** Summary of therapeutic strategies targeting CAF heterogeneity and functions in LUAD.

Therapeutic category	Representative target/agent	Main mechanism	Potential therapeutic impact in LUAD	Translational maturity	Key consideration
Phenotype-specific targeting	FAP-targeted stromal approaches	Selective depletion of FAP^+^CAFs and disruption of stromal support (107, 108)	May improve perfusion, facilitate immune-cell infiltration, and suppress tumor progression (107, 108)	Preclinical to early translational	Potential on-target/off-tumor toxicity and limited stromal specificity
Phenotype-specific targeting	CXCL14-associated transitional myCAFs	Targeting the CXCL14-associated program involved in EMT, angiogenesis, and EGFR-TKI resistance (32, 109)	May suppress metastatic stromal programs and improve sensitivity to EGFR-targeted therapy (32, 109)	Preclinical	LUAD-specific clinical validation remains limited
Dual-targeted stromal immunotherapy	Bispecific CAR-based strategies	Targeting CAF markers together with tumor antigens and stromal immunosuppressive signaling (110, 111)	May increase IFN-γ secretion and reduce Treg abundance, although evidence remains largely preclinical (110, 111)	Preclinical	Safety, antigen selection, and manufacturability remain key barriers
Functional heterogeneity targeting	MCT inhibitors/CAF metabolic targeting	Blocking lactate transport and interrupting CAF–tumor metabolic coupling (103, 113)	May weaken metabolic support for tumor survival and enhance radiosensitivity (114–117)	Preclinical	Excessive blockade may induce compensatory CAF reprogramming
Immune microenvironment remodeling	Losartan	Modulation of TGF-β-related stromal remodeling and matrix fibrosis (100, 107)	May improve vascular normalization and enhance penetration of drugs and immune cells (100, 107)	Translational interest	Evidence in LUAD remains indirect and context-dependent
Immune microenvironment remodeling	Abemaciclib	Induction of a senescence-like stromal state in parallel with tumor-cell cycle arrest (118)	May alter stromal composition and reshape the immune microenvironment, although direct evidence in LUAD-associated CAFs remains limited (118, 119)	Preclinical to translational interest	Senescence-associated effects may be context-dependent

## 7 Summary and outlook

As key regulators of the LUAD tumor microenvironment, CAFs make up a complex and heterogeneous stromal network involved in tumor growth, invasion, immune escape, and therapeutic resistance. Given this functional diversity, broad and non-selective depletion of CAFs is unlikely to be an optimal strategy. Future interventions should place more emphasis on “precise regulation”, with the goal of identifying and targeting pro-tumor CAF subpopulations while preserving or restoring CAF phenotypes with anti-tumor potential, and combining this strategy with existing treatment approaches in a more deliberate way.

A clearer understanding of how CAFs interact with other components of the TME will provide an important basis for the design of new combination therapies. Systematic characterization of the molecular features of CAFs and their dynamic responses under therapeutic pressure may help define functional regulatory targets more accurately. On this basis, combining CAF-targeted agents with standard therapies may offer a practical way to overcome resistance and improve clinical outcomes in LUAD.

Undeniably, the clinical translation of CAF-directed interventions is still hindered by fundamental obstacles, particularly spatial-temporal heterogeneity and the scarcity of reliable predictive biomarkers. Nevertheless, continuous advancements in basic research provide the necessary foundation to incrementally bridge the gap between biological discovery and clinical application. Overcoming these translational hurdles will be pivotal to refining stromal regulation and improving therapeutic outcomes in LUAD, ultimately transforming stroma-targeted precision medicine from a conceptual framework into a clinical reality.
